# Enhancing Chimeric Antigen Receptor T‐Cell Generation via Microfluidic Mechanoporation and Lipid Nanoparticles

**DOI:** 10.1002/smll.202410975

**Published:** 2025-03-19

**Authors:** Jianhua Lim, Daniel Oh, Makayla Cheng, Uday Chintapula, Shujing Liu, David Reynolds, Xiaogang Zhang, Yumeng Zhou, Xiaowei Xu, Jina Ko

**Affiliations:** ^1^ Department of Bioengineering University of Pennsylvania Philadelphia PA 19104 USA; ^2^ Department of Pathology and Laboratory Medicine Perelman School of Medicine University of Pennsylvania Philadelphia PA 19104 USA

**Keywords:** CAR‐T, immunotherapies, lipid nanoparticles, microfluidics, transfection

## Abstract

Chimeric antigen receptor (CAR)‐T cell therapy has revolutionized cancer treatment by engineering patients' T cells to specifically target cancer cells. Traditional CAR‐T cell manufacturing methods use viral transduction to integrate CAR genes into T cells, but this can cause severe side effects and immune reactions and is costly. To overcome these challenges, non‐viral methods, such as plasmid DNA (pDNA) transfection, are being explored. Here, a high‐throughput intracellular delivery platform that integrates microfluidic mechanoporation with lipid nanoparticle (LNP)‐based delivery, LNP + Squeeze, is introduced. This system enhances pDNA transfection efficiency in T cells while maintaining cell viability compared to other non‐viral transfection methods like electroporation. This platform successfully engineers CAR‐T cells using primary human T cells with a high transfection efficiency and demonstrates potent cytotoxicity against melanoma cells. This approach offers a promising, cost‐effective, and scalable alternative to viral methods, potentially improving the accessibility and efficacy of CAR‐T cell therapies.

## Graphical abstract

A high‐throughput intracellular delivery platform that integrates microfluidic mechanoporation with lipid nanoparticle‐based delivery, LNP + Squeeze, is introduced. The platform can engineer CAR‐T cells using primary human T cells with a high transfection efficiency.

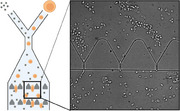

## 1 Introduction

CAR‐T cell therapy has transformed cancer treatment by engineering patients' T cells to precisely target and eradicate cancer cells.^[^ ^1^, ^2^ ^]^ The current gold standard for generating CAR‐T cells relies on viral transduction to integrate specific CAR genes into the patient's T cells’ genome.^[^ ^3^ ^]^ As of 2024, all six Food and Drug Administration (FDA)‐approved CAR‐T cell therapies utilize lentiviral and gamma retroviral vectors to deliver CAR genes into T cells. Despite exhibiting a high transduction efficiency of up to 70%, viral‐based transduction can trigger severe adverse effects and provoke unwanted immune responses such as cytokine release syndrome (a systemic inflammatory response that can be caused by many other immunotherapies, infections, and autoimmune diseases), neurologic toxicities, prolonged cytopenias, and even cancer development.^[^ ^3^, ^4^, ^5^, ^6^ ^]^ Recently, the FDA issued a boxed warning for “T cell malignancies following treatment with B‐cell maturation antigen (BCMA)‐directed or CD19‐directed autologous CAR‐T cell immunotherapies.”^[^ ^7^ ^]^ This highlights the need for alternative methods of manufacturing CAR‐T cells without using viral vectors.

Recently, different non‐viral techniques, such as the intracellular delivery of messenger RNA (mRNA) or plasmid DNA (pDNA), have been explored as alternatives to viral transduction for inducing transient cellular expressions.^[^ ^8^, ^9^, ^10^ ^]^ The expression of mRNA is short‐lived due to its rapid translation into protein followed by degradation.^[^ ^11^ ^]^ In contrast, pDNA expression persists longer than mRNA, as it can induce transgene expression in the host cells. However, it remains transient compared to viral transduction methods, as not all pDNA will be integrated into the host cell's genome, and the remaining pDNA will eventually be lost as eukaryotic cells lack the machinery to propagate plasmids, resulting in reduced expression with every generation of dividing cells.^[^ ^12^ ^]^ Despite this, these methods offer the advantage of avoiding the risk of genotoxicity associated with integrating vectors into the host genome, which can trigger permanent gene expression in cells and potentially lead to long‐term autoimmune toxicities.^[^ ^13^ ^]^ While mRNA‐based transfection offers higher efficiency than plasmid‐based transfection by circumventing the need for nuclear translocation and transcription, its elevated cost poses a challenge for cost‐effective strategies.^[^ ^14^, ^15^ ^]^

Intracellular pDNA delivery methods can be broadly categorized into membrane disruption‐mediated and carrier‐mediated strategies.^[^ ^16^, ^17^ ^]^ Electroporation, the gold standard for hard‐to‐transfect primary cells, achieves high transfection efficiencies but often causes significant cell death due to the high electrical currents required to generate pores in both cell and nuclear membranes.^[^ ^18^, ^19^, ^20^, ^21^, ^22^ ^]^ Microfluidic mechanoporation offers an alternative by squeezing cells through narrow channels to create transient pores, maintaining good viability but with lower pDNA transfection efficiency, especially in primary human T cells.^[^ ^23^, ^24^, ^25^, ^26^, ^27^ ^]^ Notably, a microfluidic cell stretching platform achieved ≈20% transfection efficiency in human mesenchymal stem cells, while another microfluidic droplet cell mechanoporation platform demonstrated ≈75% transfection efficiency in K562 cells.^[^ ^28^, ^29^ ^]^ While these studies represent significant progress, especially given the limited number of works demonstrating successful pDNA transfection via mechanoporation, there remains no comparable study that has achieved effective pDNA transfection in primary human T cells, which are among the most challenging to transfect using non‐viral methods.^[^ ^30^ ^]^

Conversely, carrier‐mediated methods like polyethylenimine (PEI) and lipofectamine use positively charged nanocarriers to facilitate pDNA delivery, but their high cytotoxicity makes them less suitable for clinical use.^[^ ^31^, ^32^, ^33^, ^34^, ^35^ ^]^ Furthermore, using PEI and lipofectamine for transfection in primary cells also results in low transfection efficiency.^[^ ^12^ ^]^ Lipid nanoparticles (LNPs) are gaining attention for CAR‐T cell manufacturing due to their high transfection efficiency and cell viability.^[^ ^8^, ^36^, ^37^, ^38^ ^]^ LNP‐based pDNA transfection remains less common than mRNA approaches due to the relatively low transfection efficiencies in primary human T cells.^[^ ^39^ ^]^ This can be attributed to the larger size of pDNA compared to the more commonly used mRNA and small interferring RNA (siRNA) payloads. Therefore, this reduces the pDNA payload release efficiency during endosomal escape – a crucial process that already imposes a significant bottleneck for mRNA/siRNA‐based LNP delivery, with release efficiencies as low as 1–2%.^[^ ^40^ ^]^ This underscores the need for improved methods to reduce the wastage of patient‐collected cells.

**Figure 1:**
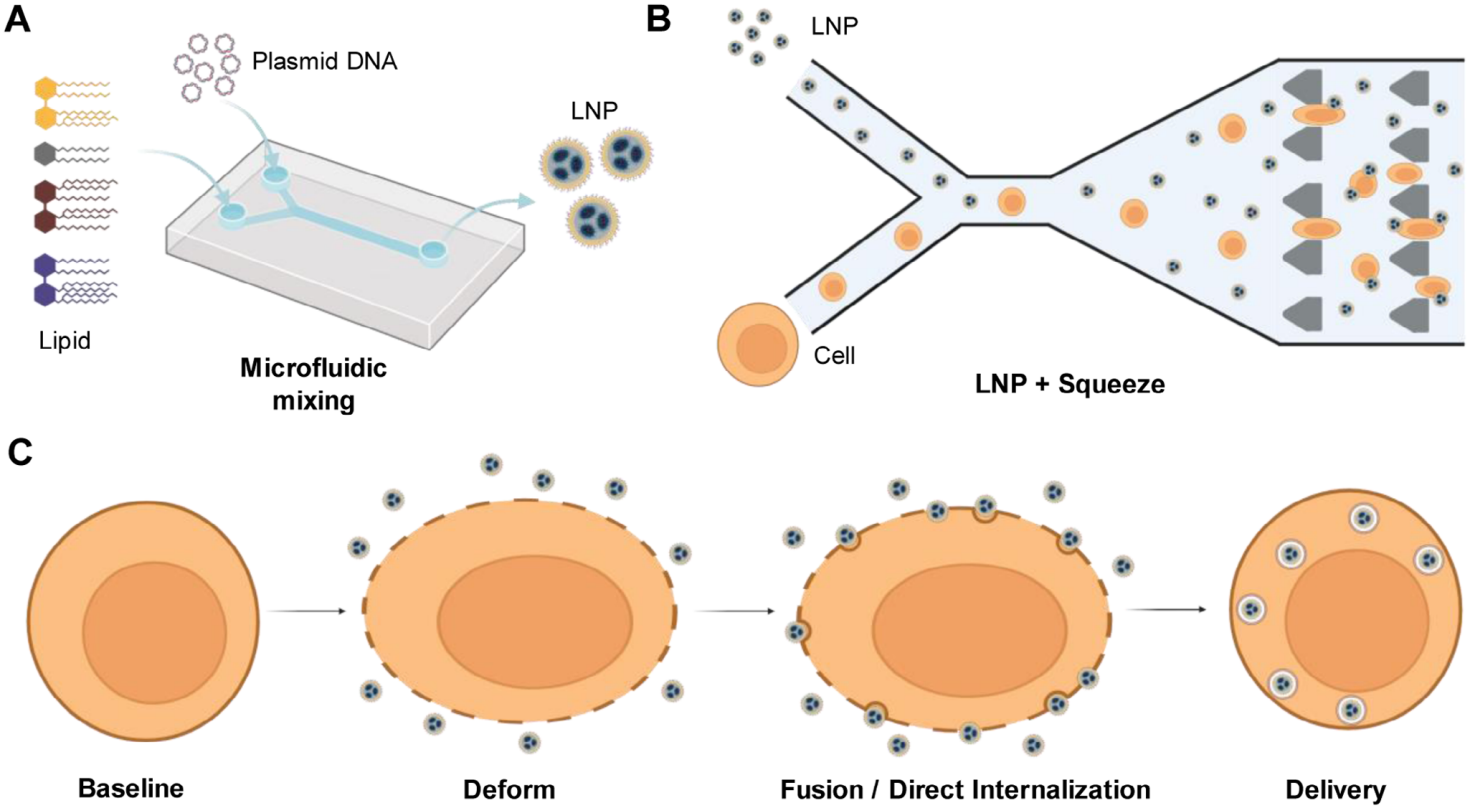
LNP + Squeeze platform design and workflow. A) Schematic of LNP synthesis using a herringbone mixer microfluidic device. B) Schematic of the microfluidic squeeze device showing the flow of LNPs and cells. C) Schematic illustrating the intracellular delivery mechanism of pDNA‐loaded LNPs through mechanoporation.

Here, we present a rapid and highly efficient intracellular delivery system that combines microfluidic‐controlled mechanoporation with LNP‐based delivery mechanisms into a single intracellular delivery platform (LNP + Squeeze) that retains high cell viability (**Figure** **1**). We performed a screening of a pDNA‐loaded LNP library to identify the optimal lipid composition for our LNP formulation for downstream transfection studies. Our cell squeezing microfluidic platform was designed and optimized for high throughput to maximize transfection yields and to significantly reduce the risk of cell clogging. We achieve this by utilizing highly parallelized squeezing regions with gaps instead of channels, featuring multiple gaps per row and multiple rows within a compact microfluidic device. To demonstrate the feasibility of our LNP + Squeeze platform, we transfected primary human T cells with CAR‐DR5‐4‐1BB‐CD3ζ expressing pDNA (CAR pDNA) that encodes for the CAR gene, alongside death receptor 5 (DR5), TNF receptor superfamily 9 (4‐1BB, commonly known as CD137), and T‐cell surface glycoprotein CD3 zeta chain (CD3ζ). DR5 receptors are commonly found overexpressed in cancer cells, such as melanoma cells, while 4‐1BB (CD137) is critical in CARs as it provides powerful costimulatory signals upon ligation.^[^ ^41^, ^42^, ^43^ ^]^  The CD3ζ signaling domain allows the CAR to mimic the natural T cell receptor (TCR) signaling and initiate T cell activation.^[^ ^44^ ^]^ With our technology, we successfully generated CAR‐T cells with high transfection yield and potent cytotoxicity when co‐cultured with various melanoma cell lines.

## 2 Experimental Section

### 2.1 LNP Synthesis and Characterization

Plasmid‐encapsulated lipid nanoparticles (LNPs) were synthesized by mixing an ethanolic phase containing lipid formulations with an aqueous phase of pDNA using a herringbone mixer microfluidic device at a 1:2.5 volume ratio with syringe pumps (Harvard Apparatus) as previously reported.^[^ ^45^ ^]^ The ionizable lipids, including C12‐200 (Cayman Chemical), 1,2‐dioleoyl‐3‐dimethylammonium‐propane (DODAP, Avanti Polar Lipids), and DLin‐MC3‐DMA (Cayman Chemical), as well as the cationic lipid 1,2‐dioleoyl‐3‐trimethylammonium‐propane (DOTAP, Avanti Polar Lipids), were prepared in ethanol at 10 mg mL⁻^1^ starting concentrations. Stock lipid formulations were made by combining these ionizable and cationic lipids with 1,2‐dioleoyl‐sn‐glycero‐3‐phosphoethanolamine (DOPE, Avanti Polar Lipids), cholesterol (Avanti Polar Lipids), and 1,2‐dimyristoyl‐sn‐glycero‐3‐phosphoethanolamine‐N‐[methoxy(polyethylene glycol)‐2000] (ammonium) (C14‐PEG 2000, Avanti Polar Lipids) in ethanol at 10 mg mL⁻^1^ individual concentrations, at predetermined molar ratios (**Tables** **1** and **2**). The aqueous phase containing pDNA was prepared in 10 mm citrate buffer, pH 3.0 (Thermo Scientific Chemicals). The plasmids used were 3705 bp pCMV‐GFP (Altogen Biosystems), 9309 bp CAR‐DR5‐4‐1BB‐CD3ζ, and an in‐house modified 8422 bp GFP plasmid. Post‐synthesis, the LNPs underwent a 2‐h dialysis period against PBS in Spectrum Spectra/Por Float‐A‐Lyzer G2 300 kDa MWCO (Fisher Scientific). The dialyzed LNPs were sterilized by passing through 0.22 µm Pall Acrodisk sterile filters with RC membrane (VMR International LLC) and stored at 4 °C.

**Table 1:** Ionizable/cationic lipid selection table.

LNP	Molar Ratio [%]				Characterization				
	Ionizable/Cationic lipid	DOPE	Cholesterol	C14‐PEG 2000	Diameter [nm]	PDI	Zeta [mV]	pDNA [ng µL⁻^1^]	E.E. [%]
C12‐200	35	42.5	20	2.5	178.03 ± 3.06	0.120 ± 0.029	13.77 ± 0.31	59.71 ± 1.75	90.19 ± 0.18
DLin‐MC3‐DMA	35	42.5	20	2.5	185.93 ± 2.46	0.055 ± 0.009	− 3.88 ± 0.11	57.91 ± 6.26	97.19 ± 0.31
DODAP	35	42.5	20	2.5	175.83 ± 3.58	0.208 ± 0.020	− 7.45 ± 0.07	22.66 ± 2.80	87.87 ± 1.37
DOTAP	35	42.5	20	2.5	133.90 ± 1.85	0.139 ± 0.019	12.70 ± 0.46	21.87 ± 0.40	95.95 ± 0.04

**Table 2:** C12‐200 lipid composition selection table.

LNP	Molar Ratio [%]			
	C12‐200	DOPE	Cholesterol	C14‐PEG 2000
LNP 1	35	32.5	30	2.5
LNP 2	35	37.5	25	2.5
LNP 3	35	42.5	20	2.5
LNP 4	35	47.5	15	2.5

The hydrodynamic diameter, polydispersity index (PDI), and zeta potential of the LNPs were characterized using the Zetasizer Nano ZS‐90 machine (Malvern Instrument) by dynamic light scattering. The encapsulation efficiency (EE) was quantified using a PicoGreen DNA quantification assay after lysing the LNPs in 2% Triton‐X for 10 min, with fluorescence measurements obtained using a Spark multimode microplate reader (Tecan Life Sciences). Transmission electron microscopy (TEM) was performed on the optimized LNP to analyze the structure using the JEOL 1010 electron microscope operated at 80 kV at the Electron Microscopy Resource Lab (EMRL), Perelman School of Medicine, University of Pennsylvania.

### 2.2 Microfluidic Device Fabrication

Both the microfluidic squeeze device and the herringbone mixer microfluidic device were fabricated using standard photolithography and soft lithography techniques at the Singh Center for Nanotechnology at the University of Pennsylvania. The microfluidic design patterns were created in AutoCAD (Autodesk, 2024) and patterned on chrome photomasks using the Heidelberg DWL 66+ laser lithography tool (Heidelberg Instruments). For the microfluidic squeeze device, silicon molds with a height of 25 µm were fabricated using the negative photoresist SU‐8 2015 (Kayaku Advanced Materials). In contrast, the herringbone mixer microfluidic device required a two‐layer photolithography process due to differing channel and herringbone structure heights (Figure S2A, Supporting Information). Initially, the SU‐8 3050 negative photoresist (Kayaku Advanced Materials) was used to achieve a channel height of 80 µm. This was followed by a second photolithography step using the same SU‐8 3050 photoresist to create the 50 µm high herringbone structures. Polydimethylsiloxane (PDMS, Dow Corning) at 10:1 base:crosslinker ratio was cast on the silicon mold, degassed, and baked for at least 2 h in a 65 °C oven before being peeled and hole‐punched for inlets and outlets. The PDMS device was then bonded to glass slides using a plasma cleaner (Sigma Aldrich) and baked in a 65 °C oven overnight. Prior to usage, the microfluidic device was flushed with 70% ethanol, followed by cell media specific to the cells to be transfected.

### 2.3 Cell Culture

Jurkat, A375, WM9, and WM35 cells were cultured using standard protocols in RPMI 1640 medium (Invitrogen), supplemented with 10% fetal bovine serum (FBS, Corning) and 1% penicillin‐streptomycin (Pen‐Strep, Invitrogen). HEK293T cells were cultured using standard protocols in DMEM medium (Thermo Fisher), supplemented with 10% FBS and 1% Pen‐Strep. All cells were maintained at 37 °C in a humidified atmosphere with 5% CO_2_.

### 2.4 Intracellular Delivery Procedure

For the optimization of the microfluidic mechanoporation squeeze device operating parameters, cells were prepared at a concentration of 1 × 10^6^ cells/mL and mixed with 300 µg mL⁻^1^ of 4 kDa FITC‐Dextran (Sigma‐Aldrich) in culture media. This mixture was delivered into the microfluidic device at desired flow rates using syringe pumps. After squeezing, the cells were diluted in PBS to a total volume of 25 mL, spun down, and the supernatant was removed to ensure complete washing away of untransfected 4 kDa FITC‐Dextran. For positive controls of transfection, cells were treated with 5 µg of pDNA per 1 × 10^6^ cells using different methods: electroporation, lipofection, and polyethylenimine (PEI) treatment. Electroporation was performed using the Neon NxT Electroporation System (Invitrogen) with three pulses at 1150 V. For lipofectamine transfection, pDNA was mixed with Lipofectamine 3000 (Invitrogen) and added to the cells according to the manufacturer's instructions. For PEI transfection, PEI MAX (Polysciences) reagent was added to pDNA at a ratio of 6.5 µL of 1 mg mL⁻^1^ PEI MAX reagent per 1 µg of pDNA and incubated for 5 min before being added to the cells. For LNP‐based transfection, pDNA‐loaded LNPs were added to the cells at the desired pDNA concentration. For LNP + Squeeze intracellular delivery, cells mixed with LNPs were delivered into the microfluidic device as described above. After processing, the cells were collected and added into fresh culture media.

### 2.5 Housekeeping and Innate Immunity Gene Assessment

Jurkat cells were squeezed and cultured for 24 h, with unsqueezed cells serving as a control. After 24 h, RNA was extracted from both conditions using TRIzol Reagent (ThermoFisher) and labeled with GlycoBlue Coprecipitant (ThermoFisher). Genomic DNA was removed with a DNase treatment, using 2U of Turbo DNase (ThermoFisher) at 37 °C for 30 min, followed by the addition of DNase inactivation reagent for 5 min at room temperature. The iScript cDNA Synthesis Kit (Bio‐Rad) was used for reverse transcription PCR (RT‐PCR) following the manufacturer's protocol, and reactions were run in a SimpliAmp Thermal Cycler (Applied Biosystems). qPCR was performed using PowerTrack SYBR Green Master Mix (Applied Biosystems) and customized primers (Integrated DNA Technologies (IDT)). A total of 40 qPCR cycles were conducted using the QuantStudio 3 Real‐Time PCR machine (Applied Biosystems). All c_t_ values were normalized against the c_t_ value of the 18s rRNA housekeeping gene prior to the calculation of fold change using the formula

(1) $$Fold\;change=2^{-\Delta \Delta c_{t}}$$

where ΔΔc_t_ is the Δc_t_ between squeezed and unsqueezed conditions after normalization.

### 2.6 Membrane Fusion Activity Using Förster Resonance Energy Transfer (FRET)

To evaluate the membrane fusion activity between LNPs and cells, FRET activity was recorded after mixing fluorescently labeled LNPs with stained cells. Jurkat cells were prepared at a concentration of 1 × 10^6^ cells/mL and stained with 5 µm 3,3′‐Dioctadecyloxacarbocyanine perchlorate (DiO, Biotium) for 30 min at 37 °C, followed by three washes to remove excess dye. The fluorescently labeled LNPs were prepared by incorporating 1 molar ratio % of 1,1′‐dioctadecyl‐3,3,3′,3′‐tetramethylindocarbocyanine (DiI, Biotium) into the lipid mix prior to LNP formation using the herringbone mixer microfluidic device. FRET activity was measured using the Spark multimode microplate reader (FRET Excitation: 460 nm; DiO Emission: 510 nm; DiI Emission: 575 nm).

### 2.7 Plasmid Localization Study

To determine the localization of intracellularly delivered pDNA, nuclei and cytosolic DNA from squeezed and unsqueezed cells were isolated and analyzed for the presence of transfected pDNA. Jurkat cells were squeezed with pDNA‐loaded LNPs and cultured for 3 days, with unsqueezed cells serving as controls. After 3 days, the Jurkat cells were harvested and washed before DNA isolation. For the isolation of cytosolic DNA, methods described by Mosley et al.^[^ ^46^ ^]^ were followed. Briefly, cells were treated with a Cytosolic Extraction Buffer to collect the cytosolic fraction. DNA was then extracted using phenol‐chloroform, followed by proteinase K and RNase A treatment to remove cytosolic proteins and RNA, respectively. For the isolation of cell nuclei, methods described by Nadelmann et al. were used.^[^ ^47^ ^]^ Cells were treated with Nuclei Isolation Buffer and Homogenization Buffer, followed by centrifugation to pellet the nuclei. TRIzol extraction was used to isolate DNA from the nuclei pellet, followed by the removal of RNA and protein. qPCR was performed on both nuclear and cytosolic DNA using methods similar to those previously mentioned, with customized primers targeting sequence‐specific regions of the pDNA.

### 2.8 Primary Human T Cell Activation

Primary T cells were activated from fresh peripheral blood mononuclear cells (PBMCs). PBMCs were purchased and isolated from HLA‐typed leukapheresis donors at the Human Immunology Core, Perelman School of Medicine at the University of Pennsylvania. Primary T cells were activated using a 1 µg mL⁻^1^ anti‐human monoclonal antibody cocktail of CD3 (OKT3 clone)/CD28 (CD28.2 clone) (eBioscience) and stimulated for 48 h. Stimulated primary T cells were harvested and cultured in RPMI 1640 medium, supplemented with 10% FBS, 1% Pen‐Strep, and 100 IU mL⁻^1^ of recombinant human IL‐2 (Peprotech) for in vitro expansion. Primary T cells were proliferated for up to 14 days post‐activation. All experiments were performed using primary T cells before 14 days of activation.

### 2.9 CAR Plasmid Construct

The CAR‐DR5‐4‐1BB‐CD3ζ pDNA was constructed as follows. The heavy and light chain sequences of the DR5 antibody were sourced from the International Immunogenetics Information System (imgt). A codon‐optimized DNA sequence encoding the DR5‐4‐1BB‐CD3ζ was designed and synthesized by IDT. This DNA fragment was then subsequently cloned into the SFG gamma retroviral vector backbone.

### 2.10 CAR‐T Cell Generation Using LNP + Squeeze Platform

For the generation of primary CAR‐T cells using the LNP + Squeeze intracellular delivery platform, primary T cells were prepared at 1 × 10^6^ cells/mL with 35 µg of pDNA‐loaded LNPs in culture media. The cell mixture was pumped at 2 mL h⁻^1^ flow rate through the microfluidic mechanoporation squeeze device and collected at the outlet. Transfected cells were added into fresh culture media and cultured for 3 days.

### 2.11 Gamma Retrovirus Packaging and Viral Transduced CAR‐T Cell Generation

Gamma retrovirus particles were produced by transfecting HEK293T cells as described by others.^[^ ^48^ ^]^ A transfection mixture was prepared by combining 3.75 µg of the retroviral vector plasmid, 3.75 µg of PegPAM plasmid, and 2.5 µg of RD114 plasmid in 500 µL of serum‐free DMEM medium. Premixed GeneJuice (MilliporeSigma) was added to the transfection mixture and incubated for 15 min at room temperature. The transfection mixture was then added to HEK293T cells and incubated for 16 h at 37 °C. Retrovirus‐containing supernatants were collected at 24 and 48 h after transfection, replacing the supernatants each time. The collected supernatants were filtered through a 0.45 µm filter (Cytiva) and stored at − 80 °C until further use.

For generating viral transduced CAR‐T cells, the collected gamma retroviral supernatant was added to Retronectin (Takara Bio Inc.)‐coated 24‐well plates and centrifuged at 4 000 g for 1 h. Activated primary human T cells were added at a concentration of 0.5 × 10^6^ cells per well and centrifuged at 1 000 g for 10 min. The cells were cultured for 2 days before being used for experiments.

### 2.12 Flow Cytometry

Before flow cytometry, harvested cells were washed and resuspended in cell staining buffer (BioLegend). CAR expression was measured by staining with AlexaFluor 488 Anti‐Human IgG, F(ab')₂ dye (Jackson ImmunoResearch Laboratories) at a 1:50 dilution. Cell viability was assessed using 7‐AAD Viability Staining Solution (BioLegend). Cells were also stained with APC anti‐human CD45 [Clone: HI30] (BioLegend) stain as a positive control. The stained cell suspension was analyzed using the BD FACSymphony A3 Lite flow cytometer (BD Biosciences) at the Penn Cytomics and Cell Sorting Shared Resource Laboratory at the University of Pennsylvania. Flow cytometry data was analyzed and plotted using FlowJo V10 Software (BD Biosciences). During flow analysis, cells were first gated to exclude cell debris and aggregates using forward scatter (FSC) area versus side scatter (SSC) area plots. Single cells were then gated using FSC‐area and FSC‐height plots, followed by positive gating of APC fluorescence to identify CD45^+^ cells. To measure viability, cells were gated to determine the percentage of 7‐AAD negative (live) and positive (dead) populations. For measuring FITC‐Dextran loading efficiency, GFP, or CAR expression, viable cells were gated to determine the percentage of cells with green fluorescence relative to the untransfected negative control cells.

### 2.13 Killing Assays Using Engineered Primary Human CAR‐T Cells

All killing assays were conducted with Luciferase Assay System (Promega Corporation). Target luciferase‐exhibiting melanoma cancer cells were seeded in 96‐well plates at 2 × 10^4^ cells per well in 50 µL of RPMI 1640 medium without FBS growth serum and incubated for 4 h to allow for complete adhesion of the target cells. Effector T cells were then seeded on the target cells at predetermined ratios in 50 µL of primary human T cell culture medium and cultured for up to 48 h. To measure cell‐associated luciferase activity, the cells were gently spun down at 1 000 rpm for 5 min to remove the supernatant, followed by a Dulbecco's phosphate‐buffered saline (DPBS, Invitrogen) wash and the subsequent removal of the wash supernatant. 45 µL of 1X Reporter Lysis Buffer (Promega Corporation), diluted in DI water, was added to each well. This was followed by one complete freeze‐thaw cycle by freezing at − 80 °C and thawing at room temperature to ensure complete cell lysis. The lysed mixture was then transferred into a Nunc White 96‐well microplate (Thermo Scientific). Then, 100 µL of Luciferase Assay System reagent was directly added into each well, and luminescence measurements were performed immediately using a Spark multimode microplate reader (Tecan Life Sciences). Luminescence readings from wells with blank media were used to set the 100% killing efficiency (maximum cell death) reference, and luminescence readings from wells with only target cells were used to set the baseline 0% killing efficiency (target cell spontaneous death) reference. The percentage killing efficiency was calculated as

(2) $$\begin{array}{ccc} & & Killing\;efficiency=\\ & & \frac{Experimental\;data-target\;cell\;spontaneous\;death}{Blank\;media\;maximum\;cell\;death-target\;cell\;spontaneous\;death}\times 100\% \end{array}$$

### 2.14 Statistical Analysis

All data are represented as mean ± standard deviation. All analyses and plots were performed using GraphPad Prism 10 (GraphPad Software Inc). Unpaired student's t‐test was used for analysis between two groups. For all tests, p values < 0.05 indicate statistical significance.

## 3 Results

### 3.1 LNP Screening and Characterization

**Figure 2:**
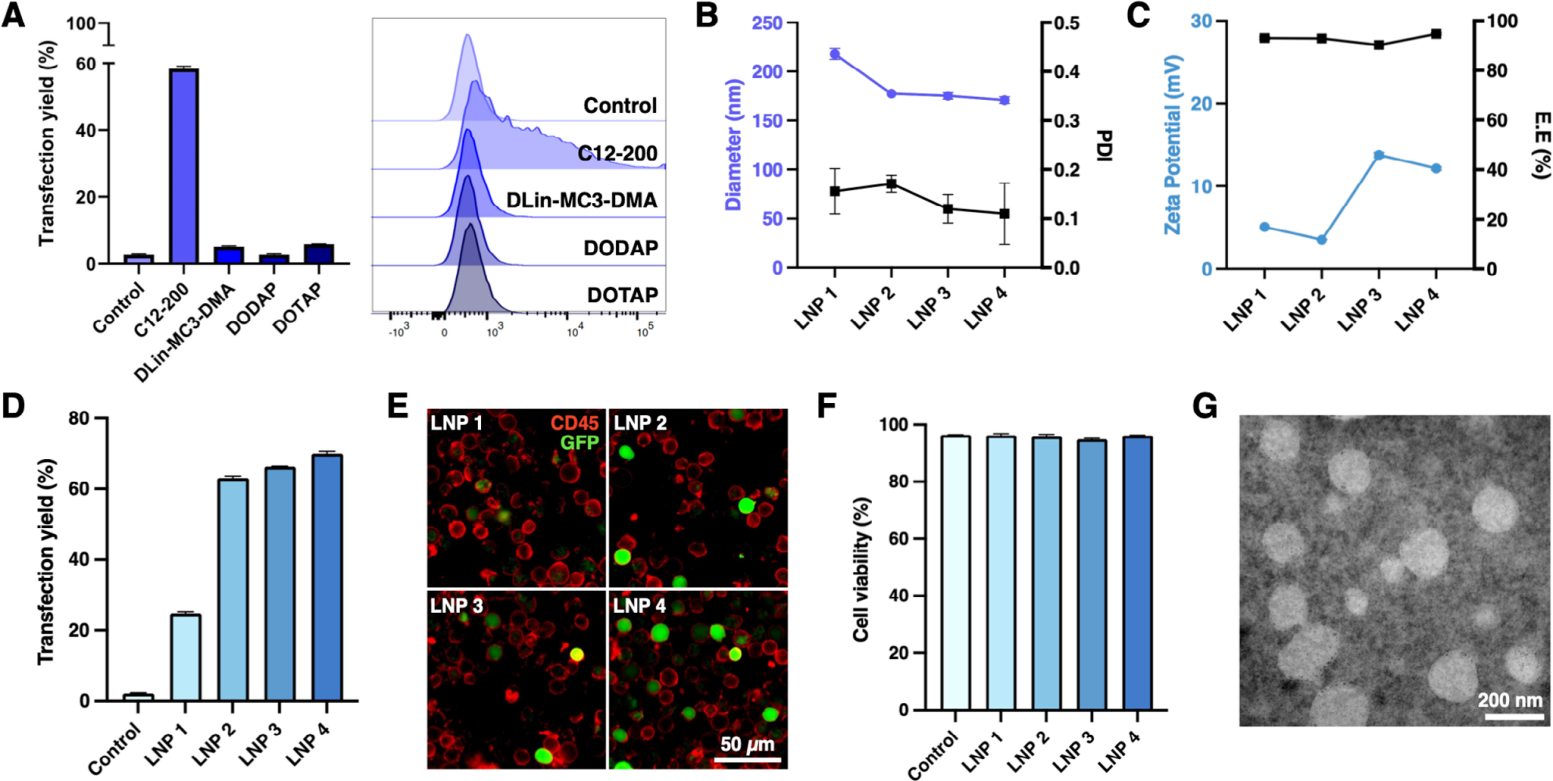
pDNA‐loaded LNP screening and characterization. A) Transfection yield and representative histograms of GFP expression in Jurkat cells 3 days after transfection with GFP pDNA‐loaded LNPs containing different ionizable/cationic lipids. B) Plots showing the diameter and polydispersity index (PDI) of different LNP formulations. C) Plots showing the zeta potential and EE of different LNP formulations. D) Transfection yield and representative histograms of GFP expression in Jurkat cells 3 days after transfection with different GFP pDNA‐loaded LNP formulations. E) Fluorescent images displaying GFP expression (green) in CD45+ Jurkat cells (red) post‐transfection. F) Cell viability of Jurkat cells following transfection. G) TEM image of pDNA‐loaded LNP formulation 4. Error bars represent the mean ± standard deviation.

We aimed to identify the optimal lipid composition for achieving the highest transfection yield while maintaining cell viability. Jurkat cells were selected as our model system as they are a human T cell leukemia line, closely mimicking our ultimate goal of transfecting primary T cells.^[^ ^19^ ^]^ To achieve this, an initial library of LNPs encapsulating GFP‐expressing pDNA (Altogen Biosystems) was synthesized using different ionizable/cationic lipids (C12‐200, DODAP, DLin‐MC3‐DMA, and DOTAP), with fixed lipid compositions of a PEG‐lipid conjugate (C14‐PEG2000), phospholipid (DOPE), and cholesterol (Table 1). These LNPs were characterized by hydrodynamic size, polydispersity index (PDI), and zeta potential. All four LNP formulations exhibited similar sizes, ranging from 134 to 186 nm, low PDI values (below 0.21), encapsulated pDNA concentrations between 22 to 60 ng µL⁻^1^, and high encapsulation efficiencies (above 88%). C12‐200 and DOTAP‐based LNPs demonstrated larger positive zeta potentials (13.8 mV and 12.7 mV, respectively), while DLin‐MC3‐DMA and DODAP‐based LNPs exhibited smaller negative zeta potentials (−3.9 mV and −7.5 mV, respectively), consistent with previous findings.^[^ ^49^, ^50^, ^51^ ^]^ Transfection yield and cell viability were assessed in Jurkat cells 3 days post‐incubation with the LNPs using flow cytometry. Among the formulations, only C12‐200 lipid‐based LNPs induced strong GFP expression (58.4%) while maintaining good cell viability (95.6%) compared to the non‐transfected control, outperforming the other ionizable/cationic LNPs (**Figure** **2**A; Figure S1, Supporting Information). Previous studies have shown that using DOTAP‐based LNPs to deliver pDNA at our DOTAP/cholesterol molar ratio typically achieves transfection efficiencies between 5–10%, with the optimal molar ratio only reaching up to 20% efficiency.^[^ ^50^ ^]^ Furthermore, DODAP and DLin‐MC3‐DMA are more commonly used for siRNA delivery rather than for pDNA due to their poor endosomal escape and DNA release characteristics.^[^ ^49^ ^]^ As expected, C12‐200‐based LNPs performed the best, consistent with recent efforts demonstrating their efficacy for pDNA delivery using LNPs.^[^ ^39^, ^51^ ^]^ Consequently, C12‐200 was selected as the ionizable lipid for downstream LNP formulation studies.

Subsequently, we screened four different DOPE and cholesterol lipid molar ratios, as described by Prazeres et al., using C12‐200 as the ionizable lipid (Table 2).^[^ ^39^ ^]^ The resulting LNPs ranged in size from 169.5 to 214.6 nm, with PDI values between 0.120 and 0.175, and high encapsulation efficiencies ranging from 90.19% to 94.70% (Figure 2B,C). Notably, increasing the DOPE molar ratio above 42.5% resulted in increased zeta potentials, consistent with previous findings.^[^ ^51^ ^]^ LNP formulations 3 and 4 exhibited significantly larger positive zeta potentials (13.8 mV and 12.1 mV, respectively) compared to the smaller positive zeta potentials of formulations 1 and 2 (5.1 mV and 3.5 mV, respectively) (Figure 2C). In Jurkat cells, the increase in DOPE molar ratio positively correlated with transfection yield, again consistent with previous reports.^[^ ^51^ ^]^ Specifically, LNP formulations 2, 3, and 4 induced strong GFP expression, with formulation 4 showing the highest transfection yield (70.1%) while maintaining excellent cell viability (96.2%) compared to the non‐transfected control (Figure 2D–F). Therefore, LNP formulation 4 was identified as the lead LNP formulation for further studies. TEM analysis revealed that LNP formulation 4 displayed a spherical morphology with an electron‐dense core (Figure 2G).

### 3.2 Microfluidic Squeeze Device Optimization

**Figure 3:**
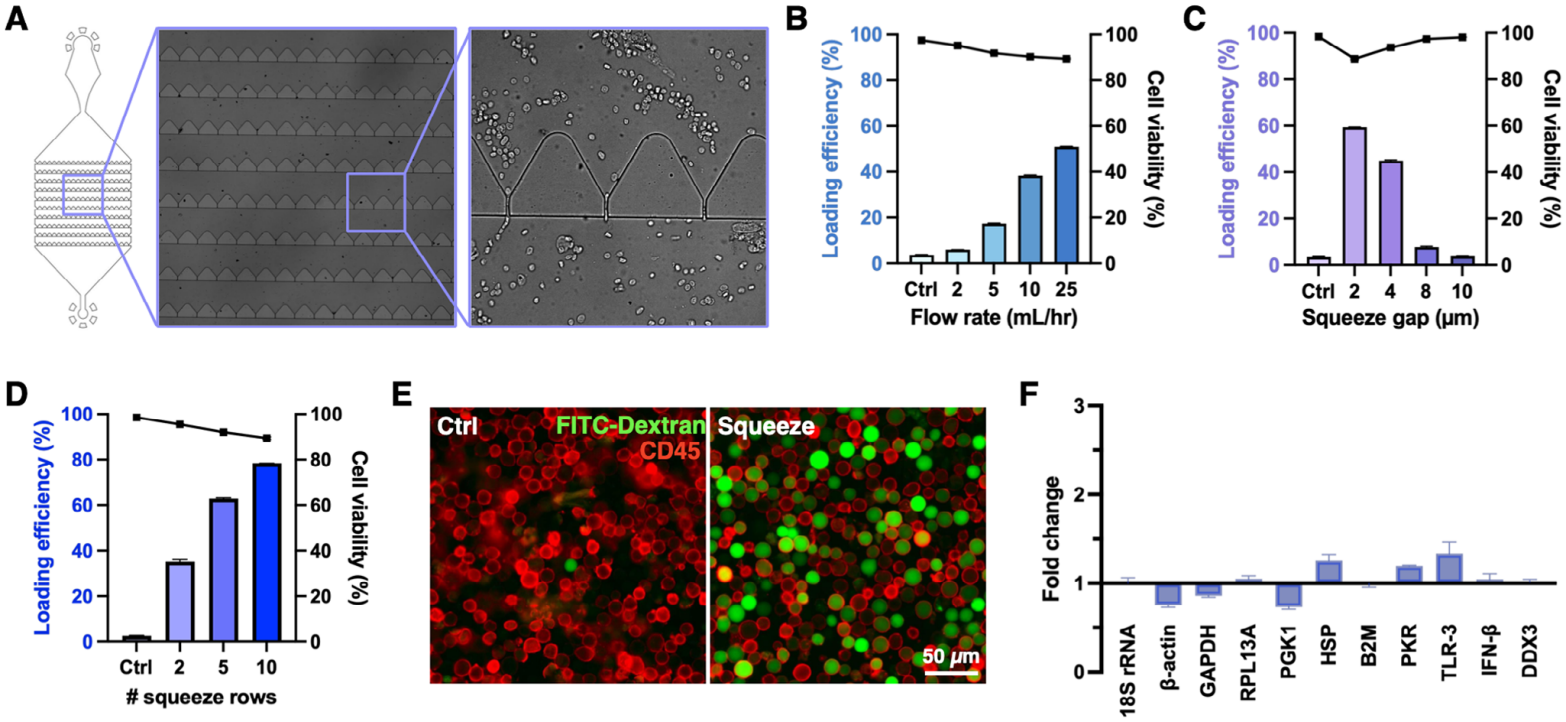
Characterization and optimization of the microfluidic squeeze device. A) Schematic of the microfluidic squeeze device with brightfield microscope images showing cell deformation as they pass through individual squeeze gaps. Loading efficiency and cell viability of Jurkat cells loaded with 4 kDa FITC‐Dextran with B) varying flow rates during the squeezing process with 2 µm squeeze gap width and ten squeeze rows, C) different squeeze gap width at 25 mL h⁻^1^ with ten squeeze rows, and D) varying numbers of squeeze rows at 25 mL h⁻^1^ with 2 µm squeeze gap width. The control is a condition where cells were incubated with FITC‐Dextran without squeeze. E) Fluorescent image of CD45+ Jurkat cells (red) loaded with 4 kDa FITC‐Dextran (green) with and without squeeze (control). F) Gene expression analysis of housekeeping and innate immunity genes in Jurkat cells 24 h post‐squeeze compared to an unsqueezed negative control. Fold changes were calculated using 18s rRNA as the reference gene. Error bars represent the mean ± standard deviation.

The intracellular delivery of payloads into cells via microfluidic mechanoporation is largely influenced by flow conditions and the design of the microfluidic channels and structures.^[^ ^27^, ^29^, ^52^ ^]^ However, most designs for microfluidic mechanoporation devices are prone to cell clogging due to the use of single squeeze channels.^[^ ^27^ ^]^ To mitigate this, we highly parallelized our squeezing regions by designing gaps instead of channels, with 20 gaps per row and multiple rows packed into a compact microfluidic device (**Figure** **3**A; Figure S2B, Supporting Information). The squeeze gap length was kept constant at 50 µm, as optimized by Kwon et al.^[^ ^53^ ^]^ To optimize gap designs and operating conditions, we loaded 4 kDa FITC‐Dextran into Jurkat cells. By varying flow rates from 2 to 25 mL h⁻^1^, we observed that the loading efficiency increased with higher flow rates compared to the unsqueezed negative control (Figure 3B). Increasing flow rates generate higher shear stress, leading to greater nucleus deformation and potentially resulting in larger transient cell pores that facilitate the exchange of cytosolic and external fluids.^[^ ^54^, ^55^, ^56^ ^]^ The loading efficiency began to plateau at flow rates above 25 mL h⁻^1^, and higher flow rates were also avoided due to the excessive internal microfluidic channel pressure that led to tubing bursts and cell loss. Given that Jurkat cells range from 10 to 16 µm, we optimized the squeeze gap widths by varying them between 2 and 10 µm, as previous reports indicate that the optimal gap widths should be between 15–30% of the cell diameter.^[^ ^27^, ^57^ ^]^ We observed that devices with 2 µm squeeze gap widths achieved the best loading efficiency without compromising cell viability (Figure 3C). When varying the number of parallel squeeze rows, we found that ten squeeze rows exhibited the best loading efficiency (Figure 3D). As cell viability did not significantly decrease even with up to ten rows, we selected ten squeeze rows to accommodate harder‐to‐transfect cells and larger payloads. Using the optimized parameters (2 µm squeeze gap and ten squeeze rows), we demonstrated high cell recovery efficiencies post‐squeezing (84.36% and 88.67% when operated at 2 mL h⁻^1^ and 25 mL h⁻^1^ respectively) (Figure S3, Supporting Information).

Jurkat cells loaded with 4 kDa FITC‐Dextran under optimized conditions (2 µm gap, 25 mL h⁻^1^, and ten rows) exhibited excellent loading efficiency (78.4%) that is comparable to previous studies, while retaining great cell viability (89.5%) and maintaining similar phenotypic characteristics to unsqueezed cells (Figure 3E).^[^ ^27^ ^]^ To ensure negligible effects of cell squeeze on cell perturbation, we compared the expression of seven housekeeping genes and four innate immunity genes between squeezed and unsqueezed cells 24 h post‐squeezing.^[^ ^58^, ^59^, ^60^, ^61^, ^62^, ^63^, ^64^, ^65^, ^66^ ^]^ qPCR analysis revealed no significant differences in gene expression (unpaired t‐test; p = 0.998). Using 18s rRNA as a housekeeping gene to calculate fold change, each gene remained close to 1, indicating negligible differences between squeezed and unsqueezed cells (Figure 3F).

### 3.3 LNP + Squeeze Enabled Intracellular Delivery

**Figure 4:**
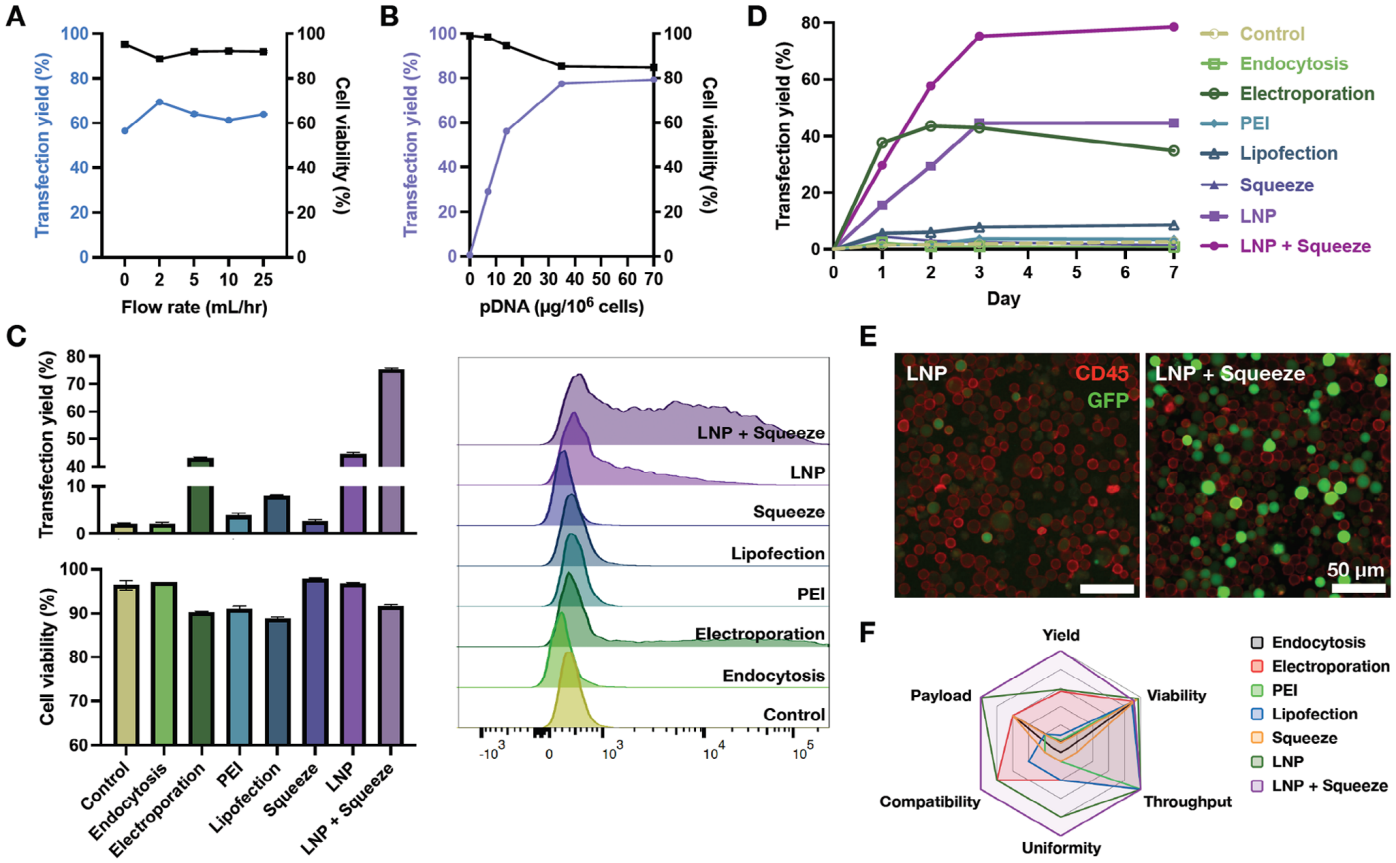
Intracellular delivery of GFP‐pDNA into Jurkat cells using the LNP + Squeeze intracellular delivery platform. A) Transfection yield and representative histograms of GFP expression in Jurkat cells 3 days post‐transfection with GFP pDNA‐loaded LNPs using the LNP + Squeeze method with the optimal LNP formulation 4. B) Transfection yield and cell viability of Jurkat cells following incubation with GFP pDNA‐loaded LNP formulation 4 at varying dosages, corresponding to different concentrations of encapsulated GFP pDNA. C) Transfection yield, cell viability, and representative histograms of GFP expression in Jurkat cells 3 days post‐transfection with GFP pDNA, comparing different transfection methods. The control is a condition where cells are cultured without the presence of GFP pDNA. D) Time‐course analysis of GFP expression in Jurkat cells using various transfection methods. E) Fluorescent images of CD45+ Jurkat cells (red) post‐transfection with GFP pDNA (green), comparing LNP + Squeeze versus LNP‐only methods. F) Radar chart evaluating the transfection performance of different transfection methods.

Next, we aimed to test our hypothesis that combining LNP uptake with microfluidic mechanoporation would enhance transfection efficiencies by accelerating LNP fusion through temporary cell deformation. To our knowledge, this is the first reported instance of integrating a membrane disruption‐mediated method with a carrier‐mediated one. Previous efforts had typically focused on combining different membrane‐disruption methods, such as mechano‐electroporation and electro‐sonoporation, rather than merging membrane disruption with carrier‐mediated delivery.^[^ ^24^, ^67^, ^68^, ^69^, ^70^, ^71^ ^]^ We hypothesized that the combined LNP + Squeeze approach required re‐optimization of flow conditions to lower and gentler flow rates compared to squeeze‐only methods. This was necessary because while the delivery of FITC‐Dextran needed to be entirely intracellular, LNP‐loaded pDNA delivery required gentler flow rates to promote fusion of the LNP to the cell membrane. As anticipated, a flow rate of 2 mL h⁻^1^, unlike the previously optimized flow conditions for squeeze‐only methods, provided a slightly better transfection yield (69.47%) without substantially decreasing cell viability (**Figure** **4**A; Figure S4A, Supporting Information). This improvement may also be attributed to the increase in effective channel diameter at higher flow rates, which diminishes the squeeze effects on the cells (Figure S4B, Supporting Information).

To better understand how the LNP + Squeeze approach enhances transfection efficiency, we designed two experiments to determine whether LNPs in this method enter cells through membrane fusion or via intact passage through mechanically created nanopores. To assess membrane fusion activity, we measured FRET signals between DiI‐labeled LNPs and DiO‐labeled Jurkat cells.

Here, DiO is used as a FRET donor, while DiI is used as a FRET acceptor. In the LNP + Squeeze method, we observed an increase in the DiI/DiO fluorescence intensity ratios compared to incubating the cells with LNP only (Figure S5A, Supporting Information). This indicates that there is a higher membrane‐fusion activity by using the LNP + Squeeze method. The increase in DiI/DiO fluorescence intensity ratio was observed over the course of 3 days (Figure S5B, Supporting Information). On the other hand, to understand the role of mechanoporation in LNP uptake, we compared the transfection efficiency of smaller (131.4 nm) and larger (154.7 nm) GFP pDNA‐loaded LNPs. Since the nanopores generated by squeezing are ≈200 nm in size, they allow for passive diffusion of LNPs.^[^ ^54^ ^]^ According to the Stokes‐Einstein‐Sutherland equation, particle diffusibility is inversely proportional to its Stokes radius, leading us to hypothesize that larger LNPs would exhibit lower transfection efficiency than smaller LNPs.^[^ ^72^ ^]^ Consistent with this hypothesis, we observed a slight decrease in transfection efficiency with larger LNPs compared to smaller LNPs (78.8% vs 89.4%, respectively), suggesting that LNPs are also directly trafficked across the mechanically induced nanopores through passive diffusion (Figure S5C, Supporting Information). Therefore, we rationalize that the LNP + Squeeze method has a dual synergistic action whereby the increase in membrane fusion activity of the LNP promotes internalization of the LNPs, while the mechanically generated nanopores allow some LNP to bypass the fusion‐event for direct internalization.

Furthermore, we optimized the LNP dosage for maximum transfection yield. Transfection yield increased as we raised the initial dosage from 7 to 35 µg of pDNA in LNP per 10^6^ cells, beyond which no further increase in transfection yield was observed (Figure 4B; Figure S6, Supporting Information). To investigate the impact of plasmid size on transfection efficiency, we compared the smaller 3705 bp pCMV‐GFP pDNA and the larger 8422 bp GFP plasmid in Jurkat cells. Using electroporation, we found that the larger plasmid exhibited lower transfection yields than the smaller plasmid at both 35 and 100 µg of pDNA per 10^6^ cells (Figure S7A, Supporting Information). This result is consistent with previous findings, as larger plasmids face greater difficulty traversing cellular membrane pores, thereby reducing the overall transfection yield.^[^ ^73^ ^]^ Additionally, our data showed that transfection yield via electroporation decreased beyond 35 µg of pDNA per 10^6^ cells, likely due to pore overload that causes excessive membrane stress leading to cell death, or DNA aggregation at high concentrations that impedes translocation through the pores.^[^ ^21^ ^]^ When comparing electroporation to our LNP + Squeeze method at a high pDNA concentration of 100 µg per 10^6^ cells using the larger plasmid, the LNP + Squeeze platform achieved a significantly higher transfection yield (76.07%) compared to electroporation (34.53%), highlighting the robustness of our system (Figure S7B, Supporting Information). The effects of plasmid size on transfection yield also impacted our LNP + Squeeze platform. At a dosage of 35 µg of pDNA in LNP per 10^6^ cells, the transfection yield for the smaller pDNA reached 82.83%, while the larger pDNA achieved only 53.50% (Figure S8, Supporting Information). This difference is likely attributed to the lower GFP gene copy number in the larger pDNA batch as the pDNA concentration was normalized by weight.

Using the optimized flow condition and LNP dosage, we tested the GFP pDNA transfection efficiency of the LNP + Squeeze method on Jurkat cells compared to conventional methods such as electroporation, lipofection, and PEI transfection, as well as pDNA endocytosis‐only, squeeze, and LNP‐only methods. Three days post‐transfection, we observed that only electroporation, LNP‐only, and LNP + Squeeze methods elicited strong transfection yields, with the LNP + Squeeze method showing a significant increase compared to the other two methods (LNP + Squeeze: 75.23%, LNP: 44.53%, electroporation: 42.97%) (Figure 4C). We hypothesize that the squeezing mechanism facilitates LNP adhesion to the cell membrane by promoting the spontaneous formation of unique protein coronas, distinct from those formed in a static fluidic environment, thereby enhancing cell uptake and overall transfection efficiency.^[^ ^74^, ^75^, ^76^ ^]^ Furthermore, we confirmed that the LNP + Squeeze method allows more pDNA to localize in the cell nucleus rather than the cytoplasm, compared to LNP incubation alone, following endosomal escape (Figure S9A, Supporting Information). Transfection yields plateaued three days post‐transfection, while cell viability remained high for at least seven days (Figure 4D; Figure S9B, Supporting Information). Additionally, the LNP + Squeeze platform not only transfected more cells, but also delivered more payload per cell, as evidenced by the higher frequency of cells with stronger GFP expression compared to other methods (Figure 4E; Figure S9C, Supporting Information). Compared to other transfection methods, the LNP + Squeeze method exhibited stronger transfection yield while maintaining good cell viability (91.5%). The LNP + Squeeze also demonstrated high transfection throughput and uniformity, compatibility with different cell types, and allowed for denser payload packaging through LNP encapsulation (Figure 4F).

To further test the versatility of the LNP + Squeeze platform for transfecting various cell types, including adherent cell lines, we applied it to HEK293T cells. Unlike Jurkat cells, which are considered hard‐to‐transfect, HEK293T cells are well‐known for their ease of transfectability and high protein productivity, making them a commonly used model system.^[^ ^77^ ^]^ Using a relatively low dosage of 1 µg of pDNA per 10^6^ cells, the LNP + Squeeze platform exhibited significantly higher transfection yield compared to both LNP only and electroporation methods (LNP + Squeeze: 71.87%, LNP: 48.37%, electroporation: 8.63%) (Figure S10A, Supporting Information). Notably, electroporation required a much higher dosage of 35 µg of pDNA per 10^6^ cells to achieve near‐complete transfection (97.93%). In contrast, both LNP + Squeeze and LNP only methods required only 7 µg of pDNA per 10^6^ cells to achieve similarly high transfection yields (99.80% and 98.10% respectively). However, at this elevated dosage, cell viability decreased significantly for both LNP + Squeeze and LNP only methods (34.30% and 10.4% respectively) (Figure S10B, Supporting Information). This sharp decline in cell viability at higher pDNA concentrations likely reflects the over‐saturation of cells with transfected pDNA, which is perceived as a foreign molecule and induces cytotoxicity at elevated intracellular levels.^[^ ^73^, ^78^ ^]^

### 3.4 Primary Human CAR‐T Cell Engineering

**Figure 5:**
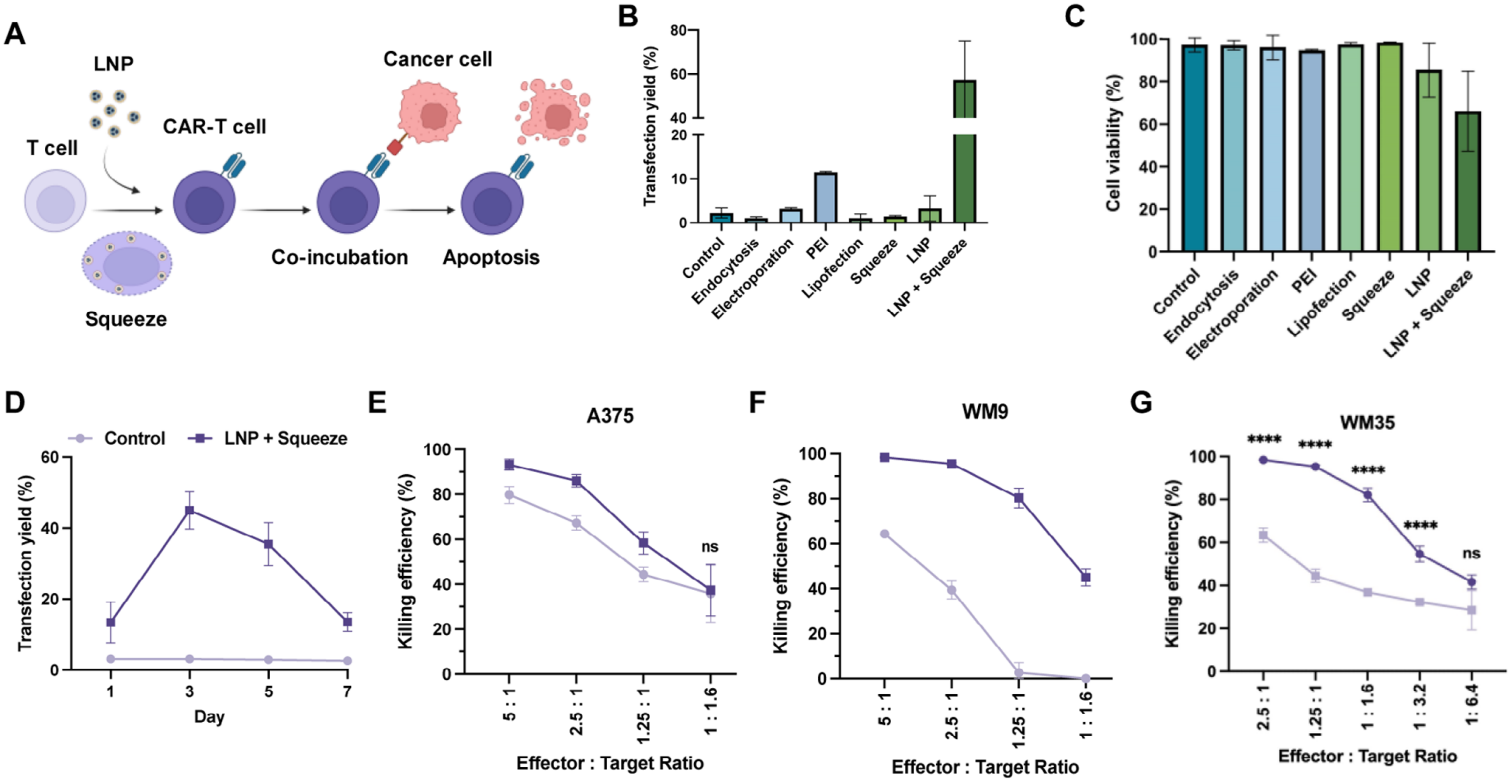
Characterization of primary human CAR‐T cells engineered using LNP + Squeeze intracellular delivery platform. A) A schematic of the transfection of primary human T cells with CAR‐pDNA, followed by the activation of effector T cells in the presence of target cancer cells. B) Transfection yield and C) viability of primary human T cells evaluated using different transfection methods 3 days after transfecting with CAR pDNA. Untreated primary human T cells were used as the control. D) Time‐course analysis of CAR expression in primary human T cells using the LNP + Squeeze intracellular delivery platform. Untreated T cells were used as the control. Killing efficiency of CAR‐T cells compared to untreated T cells control after 48 h of co‐culture with target cells: E) A375, F) WM9, and G) WM35, at various effector‐to‐target ratios. Error bars represent the mean ± standard deviation. Statistical significance is indicated by *** (P < 0.001) and **** (P < 0.0001), while “ns” denotes no significant difference.

To evaluate the potential clinical relevance of our LNP + Squeeze technology, we engineered primary human CAR‐T cells by delivering CAR pDNA using our platform, followed by the co‐incubation of the CAR‐T cells with target cancer cells (**Figure** **5**A). CAR pDNA‐loaded LNPs were synthesized using the optimized LNP formulation 4 (**Table** **3**). We compared our technology against various transfection methods on primary human T cells. Three days post‐transfection, we found that the LNP + Squeeze method achieved the highest CAR expression (57.44 ± 17.75%), as measured by flow cytometry, significantly outperforming other methods, which did not show a clear increase in CAR expression compared to the non‐transfected control (Figure 5B). However, the LNP + Squeeze method also resulted in a substantial reduction in cell viability (65.99 ± 18.71%) (Figure 5C). Interestingly, the squeeze‐only and LNP‐only transfection methods did not cause such a marked decrease in cell viability, suggesting that the viability reduction could be due to an excess of CAR pDNA translocating into the nucleus.^[^ ^73^, ^78^ ^]^ Moreover, we observed considerable variability in both transfection yield and cell viability, which can be attributed to donor‐to‐donor variability in primary human T cells which is also a common occurrence in clinical settings.^[^ ^79^ ^]^ For an optimal incubation time after transfection, we determined it to be 3 days, consistent with our previous findings in Jurkat cells (Figure 5D).

**Table 3:** CAR pDNA‐loaded LNP Characterization.

Characterization				
Diameter [nm]	PDI	Zeta [mV]	pDNA [ng µL⁻^1^]	E.E. [%]
176.97 ± 3.45	0.139 ± 0.044	4.86 ± 0.16	110.08 ± 8.94	93.86 ± 0.54

### 3.5 Specific Killing of Target Cells by Engineered CAR‐T Cells

To evaluate the functional efficacy of the engineered primary human CAR‐T cells, we co‐cultured them with various melanoma cancer cell lines at different effector‐to‐target (E:T) ratios for 48 h. When incubated with A375 cells, the CAR‐T cells showed high killing efficiencies across E:T ratios from 5:1 to 1:1.6 (Figure 5E). However, the untreated T cell control also displayed a similar trend, albeit with slightly lower efficiencies, with the difference in killing efficiency being only significant down to an E:T ratio of 1.25:1. This sensitivity could be due to A375 cells being more susceptible to molecules like perforin and granzymes secreted by activated T cells. Furthermore, A375 cells are likely to be more sensitive to activated T cells compared to naïve T cells, which express lower levels CD29 and CD69 in their quiescent state.^[^ ^80^, ^81^ ^]^ IL‐2 supplementation in the media is also known to support T cell activation and promote proliferation post‐activation, although to a lesser extent than CD3/CD28 co‐stimulation.^[^ ^82^ ^]^ To assess the susceptibility of A375 cells to minimally activated T cells, we co‐incubated unstimulated and untreated primary human T cells in media supplemented with and without IL‐2 with A375 cells for 48 h at various E:T ratios. We observed that the killing efficiency remained high, even at an E:T ratio of 1.25:1, for both with and without IL‐2 media conditions, albeit that T cells without IL‐2 media exhibited lower killing efficiencies (Figure S11, Supporting Information). This suggested that A375 cells are highly sensitive to even minimally activated T cells. However, previous studies have shown that viral vector transduction efficiencies are considerably higher in activated T cells than in naïve T cells.^[^ ^82^, ^83^ ^]^ Therefore, we selected activated T cells for CAR‐T cell generation using our platform. Despite the inherently high baseline killing efficiency of untreated activated T cells, CAR‐T cells generated via our platform demonstrated even greater killing efficiencies, underscoring the antigen‐specific cytotoxic effect of CAR‐T cells.

To further emphasize the improved killing efficiencies of the CAR‐T cells over untransfected T cells, we tested them against WM9 and WM35 cell lines, which exhibited lower killing efficiencies for the untransfected T cells. For WM9 cells, the CAR‐T cells demonstrated significantly greater killing efficiencies compared to the untransfected T cells, even at low E:T ratios where the untransfected T cells showed almost no killing (Figure 5F). At these ratios (1.25:1 and 1:1.6), the CAR‐T cells maintained substantial killing efficiencies (79.89 ± 4.01% and 47.94 ± 7.69%, respectively). Similarly, for WM35 cells, the CAR‐T cells exhibited improved killing efficiencies down to an E:T ratio of 1:3.2 (54.64 ± 3.65%) (Figure 5G). Overall, these killing assays on various melanoma cancer cells demonstrate a successful engineering of CAR‐T cells using our LNP + Squeeze platform.

To ensure that our CAR‐T cell killing efficiencies are comparable to current state‐of‐the‐art viral transduced CAR‐T cells, we co‐incubated WM9 cells with viral‐transduced CAR‐T cells for 48 h, which were normalized to the same CAR expression transfection yield as our LNP + Squeeze‐generated CAR‐T cells. We observed minimal differences in killing efficiency between LNP + Squeeze‐generated CAR‐T cells and the viral transduced CAR‐T cells at E:T ratios ranging from 5:1 down to 1:1.6, with no statistically significant differences in E:T ratios of 1.25:1 and 1:1.6 (Figure S12, Supporting Information). This demonstrates that our CAR‐T cells perform similarly to the current state‐of‐the‐art viral transduced CAR‐T cells, once normalized for the transfection yield. Therefore, our data highlights the effectiveness of our LNP + Squeeze platform in achieving comparable outcomes to conventional methods.

## 4 Discussion

Non‐viral intracellular delivery methods are revolutionizing the next generation of CAR‐T cell manufacturing, offering a safer alternative to current viral‐based transduction methods. In this study, we introduced a novel approach that combines the dual actions of membrane‐disruption mechanoporation and carrier‐mediated LNP delivery to efficiently engineer CAR‐T cells. We demonstrated that these two methods have synergistic effects on transfection efficiency when used together. By utilizing LNPs to package our payloads at high concentrations, we achieved higher delivery efficiency compared to mechanoporation alone. The versatility of LNP encapsulation, not to mention, allows for the delivery of various other types of payloads, including mRNA and siRNA, highlighting the multifaceted nature of the LNP + Squeeze platform.^[^ ^84^, ^85^ ^]^ Additionally, we believe our platform has the potential to deliver minicircle vectors encoding transposons, providing long‐term expression benefits.^[^ ^9^, ^86^, ^87^, ^88^, ^89^ ^]^ Moreover, our high‐throughput design that features parallelized rows of multiple squeeze gaps within a single microfluidic device, mitigated cell loss due to clogging which is a common issue in squeeze‐based methods. Our work is also particularly significant, as few mechanoporation‐based approaches have successfully transfected primary human T cells for CAR‐T cell generation. Those that have succeeded primarily rely on delivering mRNA payloads or ribonucleoproteins (RNPs) rather than pDNA. Notably, a microfluidic vortex shedding platform achieved transfection efficiencies of ≈60% for mRNA and 40% for RNPs in primary human T cells.^[^ ^90^, ^91^ ^]^ For CAR‐T cells generated using RNP intracellular delivery, robust NALM6 cell killing (>30%) was observed even at a low E:T ratio of 1:32. While this killing efficiency surpasses that of pDNA‐based methods, RNPs are inherently easier to deliver and express within cells, as they bypass intracellular processing steps such as transcription and translation, which are necessary for pDNA‐based approaches.^[^ ^92^ ^]^

Furthermore, our platform offers a promising solution to address the exorbitant costs associated with the current state‐of‐the‐art CAR‐T cell manufacturing and therapy. Presently, FDA‐approved CAR‐T cell therapies that rely on viral vectors for manufacturing typically cost over $400 000 USD per patient.^[^ ^93^ ^,]^ Consequently, more cost‐efficient alternatives have been explored, including mRNA‐ and DNA‐based manufacturing approaches. While mRNA‐based CAR‐T cell therapies are often delivered via in vitro transcribed (IVT) mRNA encapsulated in LNPs, a study revealed that the cost of producing IVT mRNA is nearly double that of recombinant pDNA.^[^ ^93^, ^94^ ^]^ By utilizing pDNA instead, the production cost of CAR‐T cells can be significantly reduced, with another study estimating the cost to be ≈$45 000 USD per patient.^[^ ^95^ ^]^

While our technology offers significant advantages, it also has some limitations. A deeper understanding of the specific endocytosis pathway and endocytic trafficking of the LNP + Squeeze platform is necessary to ensure that there are no long‐term effects on both the transfected T cells and the target cells. Moreover, in vivo studies of CAR‐T cell activity are essential for a more comprehensive assessment of their killing efficacy. These studies offer insights into a more complex and physiologically relevant environment by accounting for the tumor microenvironment, immune responses, and systemic effects, which are not fully captured by our in vitro assays. Although further in vivo studies are needed to determine the engraftment and efficacy of CAR‐T cells generated using our LNP + Squeeze technology, our findings suggest that the platform can potentially serve as a highly efficient and non‐toxic alternative for T cell engineering. Moreover, to further increase the efficacy of the CAR‐T cells, future studies could explore transposon‐based CAR constructs for prolonged CAR expression or mRNA‐based CAR constructs for higher translation efficiency. Future optimizations could also include refining the LNP formulation, such as incorporating antibody‐conjugated LNPs (aLNPs) for improved targeting efficiency, as well as exploring alternative payload constructs to target various solid tumors.^[^ ^96^ ^]^

## 5 Conclusions

In summary, we developed an innovative platform capable of achieving high‐throughput transfection in human primary T cells while preserving cell viability and the expression of innate immunity genes. This is accomplished through a synergistic approach that combines LNP‐mediated endocytosis with mechanoporation. To our knowledge, this is the first instance of coupling a membrane‐disruption method with a carrier‐mediated delivery system to significantly enhance transfection efficiency. Our platform has successfully demonstrated the efficient delivery of sizable plasmid payloads to hard‐to‐transfect primary human T cells for generating CAR‐T cells without relying on traditional viral vector‐based intracellular delivery methods. This approach has the potential to pave the way for more cost‐effective and scalable solutions in CAR‐T cell manufacturing and could be applied to the treatment of other diseases.

## Conflict of Interest

The authors declare no conflict of interest.

## Supporting information



Supporting Information

## Data Availability

The data that support the findings of this study are available from the corresponding author upon reasonable request.
