# Supplementary material for: Enhancing Chimeric Antigen Receptor T‐Cell Generation via Microfluidic Mechanoporation and Lipid Nanoparticles
Source: Small. 2025 Mar 19;21(17):2410975. doi: 10.1002/smll.202410975 (PMC12036559; doi:10.1002/smll.202410975)
Supplement: Supplementary file 1 — Supporting Information [file SMLL-21-2410975-s001.pdf]

NANO · MICRO
small

Supporting Information

for *Small*, DOI 10.1002/smll.202410975

Enhancing Chimeric Antigen Receptor T-Cell Generation via Microfluidic Mechanoporation and Lipid Nanoparticles

*Jianhua Lim, Daniel Oh, Makayla Cheng, Uday Chintapula, Shujing Liu, David Reynolds, Xiaogang Zhang, Yumeng Zhou, Xiaowei Xu and Jina Ko**

Supplementary Figures

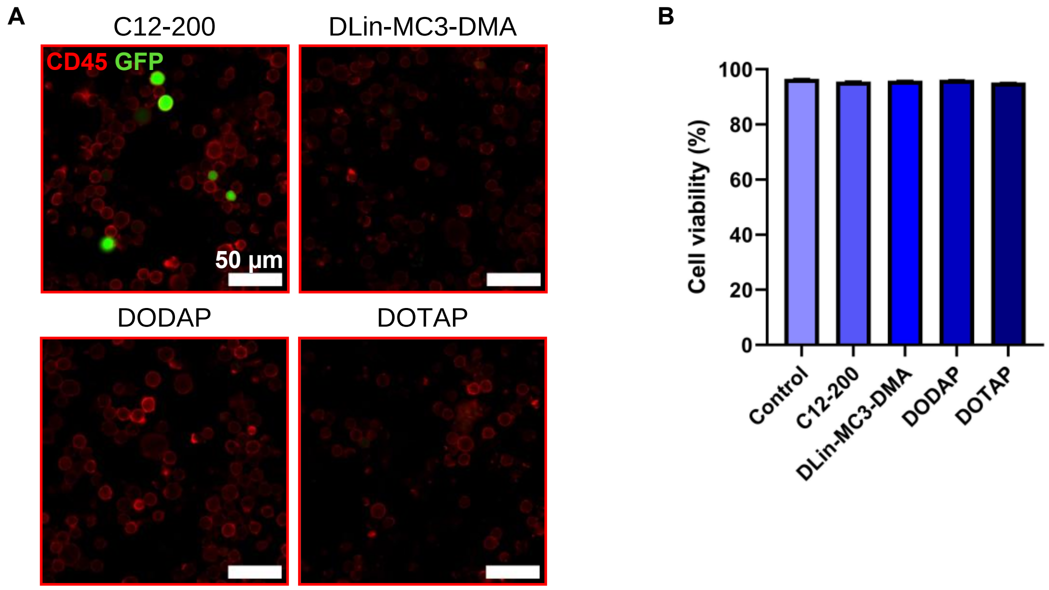

Fig S1. Jurkat cells uptake on different ionizable/cationic lipid LNP. (A) Fluorescent images of Jurkat cells expressing GFP 3 days after incubation with GFP pDNA-loaded LNPs. **(B)** Cell viability of Jurkat cells following uptake of LNPs. Error bars represent the mean ± standard deviation.

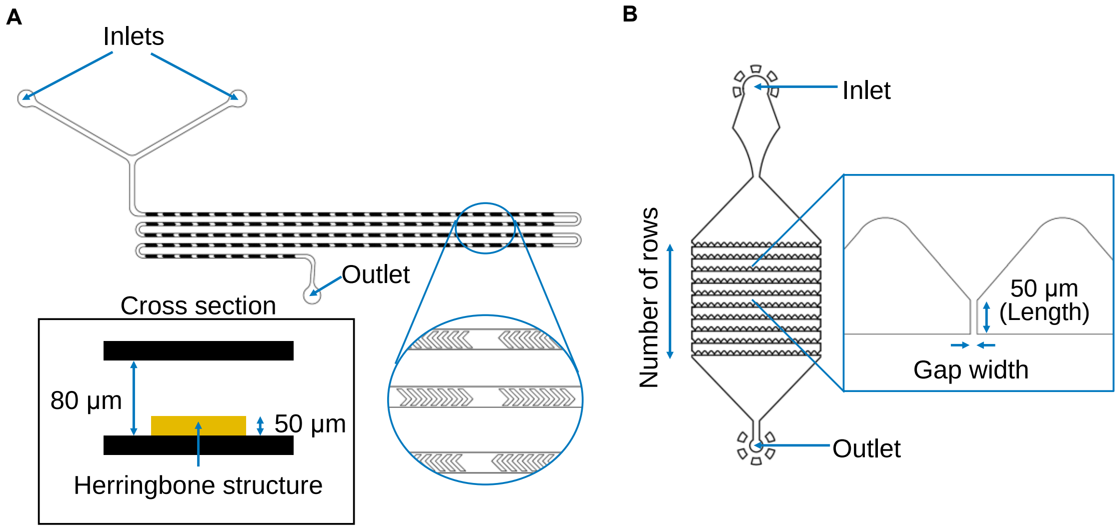

Fig S2. LNP + Squeeze platform design and workflow. (A) Schematic of the herringbone mixer microfluidic device used for LNP synthesis. **(B)** Schematic of the microfluidic squeeze device.

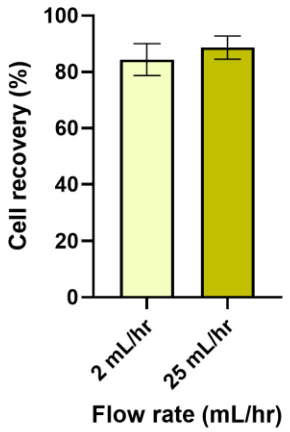

Fig S3. Jurkat cell recovery post-squeezing. Fluorescent images of Jurkat cells expressing GFP 3 days post-transfection using the LNP + Squeeze method at various flow rates.

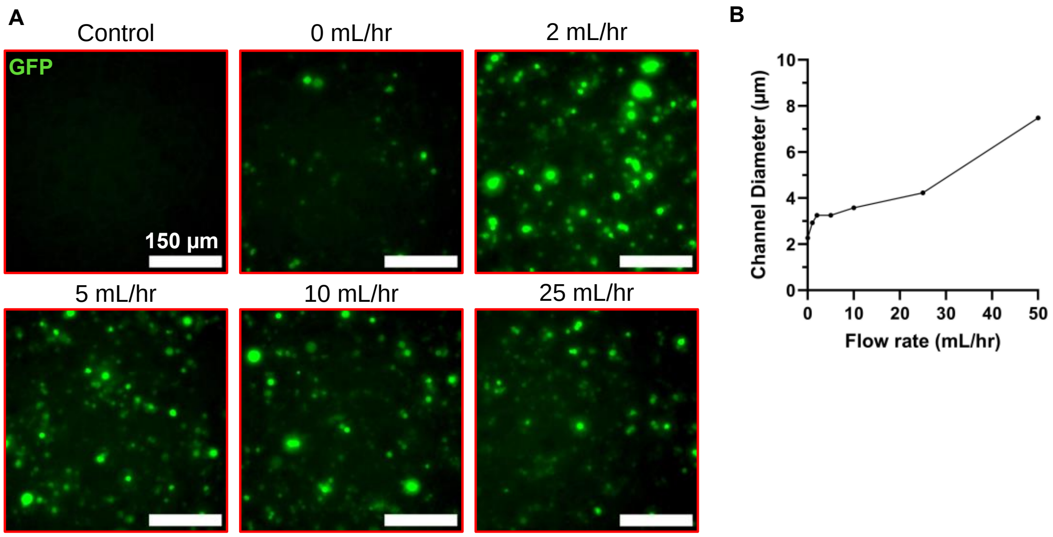

Fig S4. Jurkat transfection flow rate study for LNP + Squeeze intracellular delivery platform. (A) Fluorescent images of Jurkat cells expressing GFP 3 days post-transfection using the LNP + Squeeze method at various flow rates. **(B)** Variations in squeeze gap width across different flow rates.

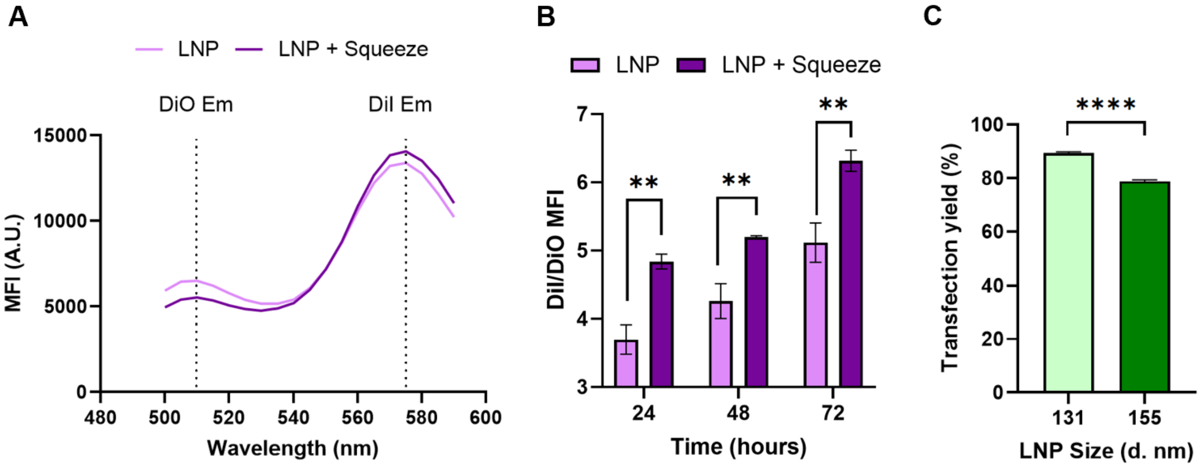

Fig. S5. Mechanistic analysis of Jurkat cell transfection using the LNP + Squeeze method. (A) Mean fluorescence intensity (MFI) comparison at specific wavelengths between LNP and LNP + Squeeze conditions after mixing DiO-labelled Jurkat cells with DiI-labelled LNP. DiO emission (Em) and DiI Em are acquired at 510 nm and 575 nm respectively. **(B)** Time-course analysis of the DiI/DiO MFI ratio for LNP and LNP + Squeeze conditions, normalized to the MFI of untreated cells. **(C)** Transfection yield of Jurkat cells using the LNP + Squeeze method with GFP pDNA-loaded LNP formulation 4, comparing two different LNP sizes. Error bars represent the mean ± standard deviation. Statistical significance is indicated by * ($P < 0.05$), ** ($P < 0.01$) and **** ($P < 0.0001$).

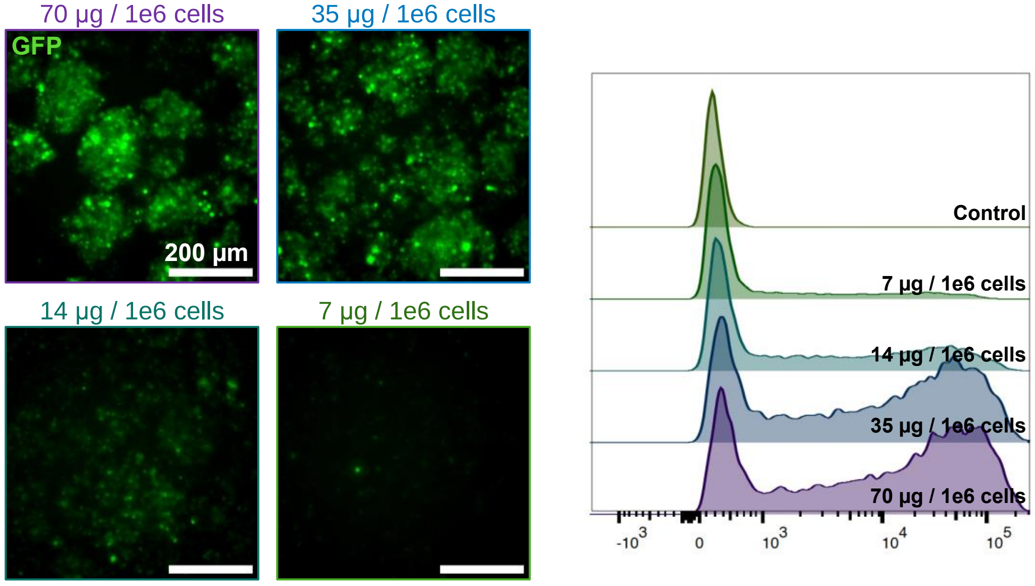

Fig S6. Jurkat transfection LNP dosage study. Fluorescent images and representative histograms of Jurkat cells expressing GFP 3 days post-transfection with the optimal GFP pDNA-loaded LNP formulation 4, incubated at varying dosages corresponding to different concentrations of encapsulated GFP pDNA.

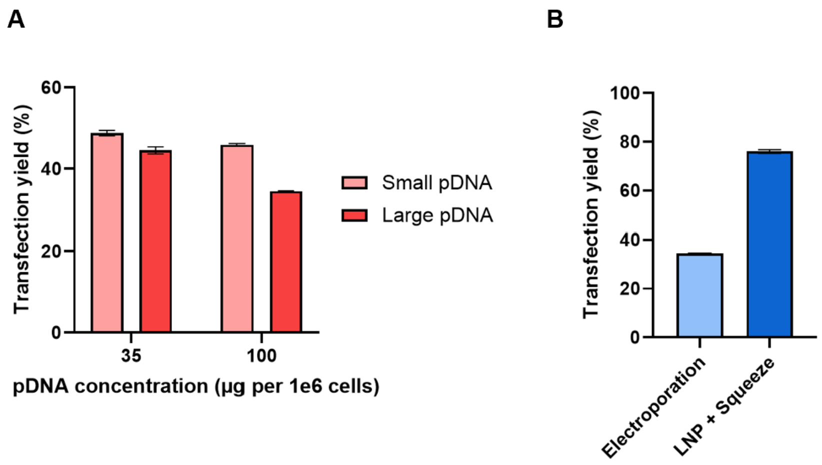

Fig S7. Jurkat transfection with different pDNA sizes. (A) Transfection yield of Jurkat cells following electroporation using smaller (3705 bp) and larger (8422 bp) GFP-expressing pDNA. **(B)** Transfection yield of Jurkat cells transfected with 100 µg of large GFP pDNA per 10^6 cells. Error bars represent the mean ± standard deviation.

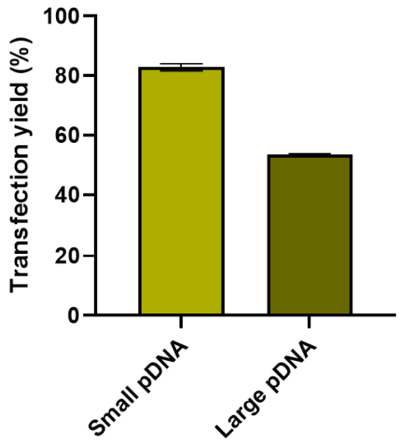

Fig S8. Transfection of Jurkat cells using LNP + Squeeze with different pDNA sizes. (A) Transfection yield of Jurkat cells transfected by LNP + Squeeze with smaller (3705 bp) and larger (8422 bp) GFP-expressing pDNA at 35 µg of pDNA in LNP per 10^6 cells. Error bars represent the mean ± standard deviation.

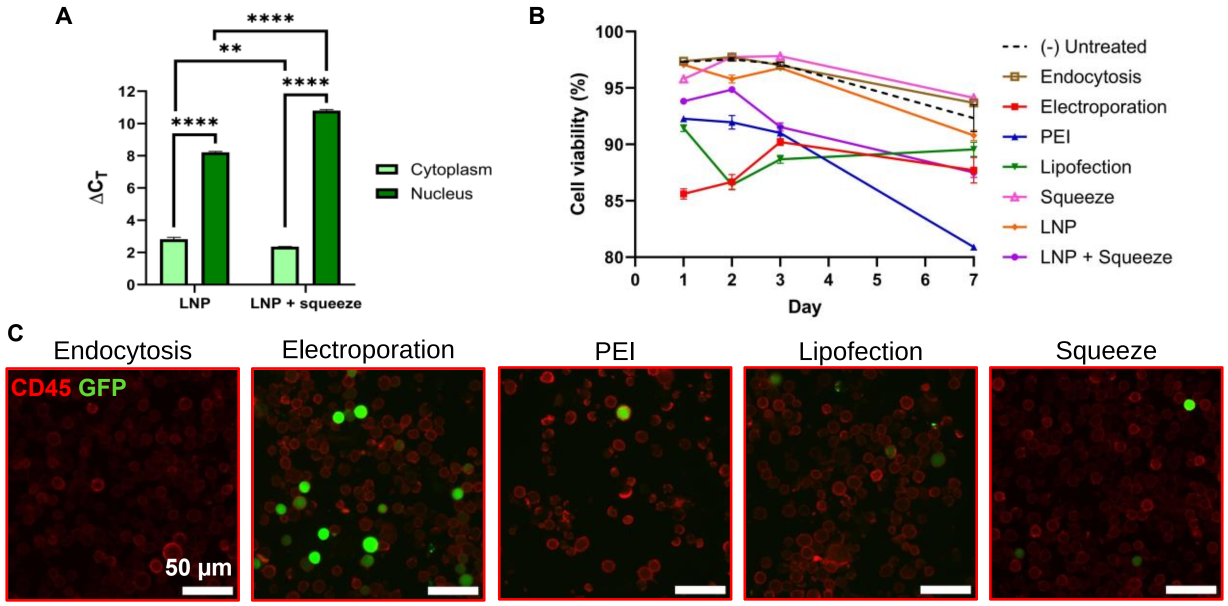

Fig S9. Jurkat cells transfected with GFP pDNA using various transfection methods. (A) Differential c_t values (Δc_t) calculated from qPCR using pDNA-specific primers between cellular DNA isolated from the cytoplasm and the nucleus. **(B)** Time-course analysis of Jurkat cell viability following transfection with GFP pDNA using various transfection methods. **(C)** Fluorescent images of Jurkat cells 3 days post-transfection with GFP pDNA using different transfection methods. Error bars represent the mean ± standard deviation. Statistical significance is indicated by ** ($P < 0.01$) and **** ($P < 0.0001$).

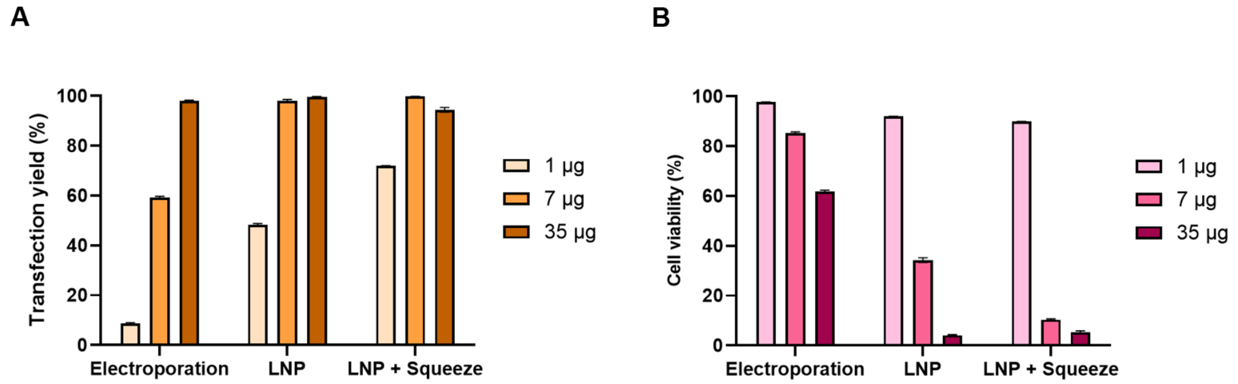

Fig S10. Transfection and viability of HEK293 cells using GFP pDNA. (A) Transfection yield of HEK293 cells at varying pDNA dosage. **(B)** Cell viability of HEK293 cells post-transfection at varying pDNA dosage. Error bars represent the mean ± standard deviation.

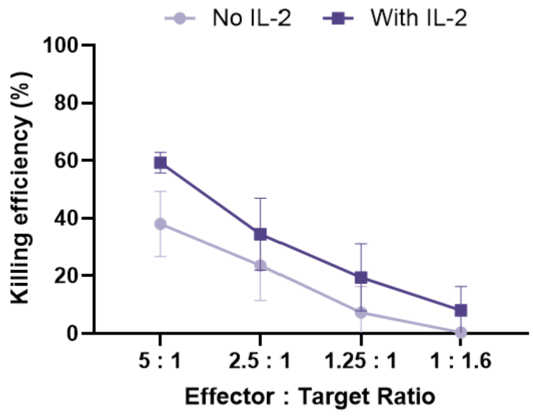

Fig S11. A375 cells are highly susceptible to killing by minimally activated T cells. Plot of killing efficiencies of minimally activated primary human T cells, either stimulated with IL-2 alone or left unstimulated, both without CD3/CD28 co-stimulation, after 48 hours of co-culture with A375 target cells at various effector-to-target ratio. Error bars represent the mean ± standard deviation.

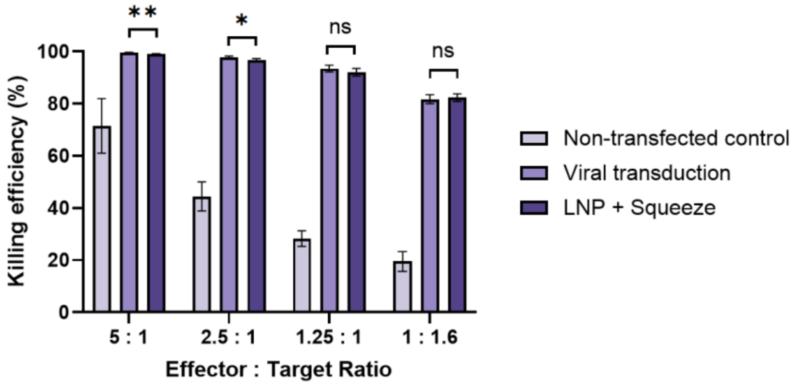

Fig S12. Functional assessment of CAR-T cells engineered using LNP + Squeeze compared to viral transduction. Plot of killing efficiencies of CAR-T cells generated using the LNP + Squeeze method versus viral transduction, compared to non-transfected T cells control after 48 hours of co-culture with WM9 target cells at various effector-to-target ratios. Error bars represent the mean ± standard deviation. Statistical significance is indicated by * ($P < 0.05$) and ** ($P < 0.01$), while "ns" denotes no significant difference.
